# Improving the Selectivity of an Osseointegrated Neural Interface: Proof of Concept For Housing Sieve Electrode Arrays in the Medullary Canal of Long Bones

**DOI:** 10.3389/fnins.2021.613844

**Published:** 2021-03-15

**Authors:** Augusto X. T. Millevolte, Aaron M. Dingle, Jared P. Ness, Joseph Novello, Weifeng Zeng, Yan Lu, Rashea L. Minor, Brett Nemke, Mark D. Markel, Aaron J. Suminski, Justin C. Williams, Samuel O. Poore

**Affiliations:** ^1^Division of Plastic Surgery, Department of Surgery, University of Wisconsin – Madison, Madison, WI, United States; ^2^Department of Biomedical Engineering, College of Engineering, University of Wisconsin – Madison, Madison, WI, United States; ^3^Department of Medical Sciences, University of Wisconsin – Madison, Madison, WI, United States; ^4^Department of Neurological Surgery, University of Wisconsin – Madison, Madison, WI, United States

**Keywords:** regenerative neural interface, neural interface, osseointegrated neural interface, amputation, amputation neuroma, neural prosthetic, prosthetic control, somatosensory evoked potential (SSEP)

## Abstract

Sieve electrodes stand poised to deliver the selectivity required for driving advanced prosthetics but are considered inherently invasive and lack the stability required for a chronic solution. This proof of concept experiment investigates the potential for the housing and engagement of a sieve electrode within the medullary canal as part of an osseointegrated neural interface (ONI) for greater selectivity toward improving prosthetic control. *The working hypotheses* are that (A) the addition of a sieve interface to a cuff electrode housed within the medullary canal of the femur as part of an ONI would be capable of measuring efferent and afferent compound nerve action potentials (CNAPs) through a greater number of channels; (B) that signaling improves over time; and (C) that stimulation at this interface generates measurable cortical somatosensory evoked potentials through a greater number of channels. The modified ONI was tested in a rabbit (*n* = 1) amputation model over 12 weeks, comparing the sieve component to the cuff, and subsequently compared to historical data. Efferent CNAPs were successfully recorded from the sieve demonstrating physiological improvements in CNAPs between weeks 3 and 5, and somatosensory cortical responses recorded at 12 weeks postoperatively. This demonstrates that sieve electrodes can be housed and function within the medullary canal, demonstrated by improved nerve engagement and distinct cortical sensory feedback. This data presents the conceptual framework for housing more sophisticated sieve electrodes in bone as part of an ONI for improving selectivity with percutaneous connectivity toward improved prosthetic control.

## Introduction

With an estimated 500 new cases every day within the United States, there is an increasing pool of amputees, living long and otherwise healthy lives (Ziegler-Graham et al., 2008; Mioton and Dumanian, 2018). A significant challenge in developing advanced motorized and sensate prosthetic devices is establishing neural interfaces that are highly selective, stable and capable of enhanced longevity (Navarro et al., 2005; Ortiz-Catalan et al., 2014; Ghafoor et al., 2017; Valerio et al., 2020). Electrode selectivity – i.e., the ability of a peripheral neural interface (PNI) to selectively stimulate or record from individual nerves – is a critical parameter inherently linked to the degrees of freedom a PNI is capable of controlling, as well as the differentiation between motor and sensory signals (Grill et al., 2009; Micera and Navarro, 2009; Horch et al., 2011; Tan et al., 2014, 2015; Cheesborough et al., 2015; Ghafoor et al., 2017). Sieve electrodes represent a type of PNI with the greatest potential for selectivity resulting from their well-spaced specificity within the nerve. These devices involve invasive nerve transection (Navarro et al., 2005; MacEwan et al., 2016; Desai et al., 2017; Ghafoor et al., 2017), and rely on robust neural regeneration through electrode transit zones, assimilating the PNI into the nerve. In the amputation setting, it may be possible to simply interface the sieve array with the residual nerve end, taking advantage of the regenerative process that would otherwise lead to neuroma formation.

Amputation neuroma is a non-tumorigenic bulbous mass that develops at the terminal end of the residual nerve, representing the innate capacity for the transected nerve to regenerate. Neuromas are a significant cause of neuropathic pain and can preclude use of a traditional socket prosthetic (Davis, 1993; Gallagher and Maclachlan, 2001). Symptomatic neuroma can be addressed utilizing a multitude of techniques including transposition into muscle and targeted muscle reinnervation (Woo et al., 2016; Mioton and Dumanian, 2018). Another proven treatment to handle neuropathic pain resulting from amputation neuromas transposes the affected nerve via a corticotomy into the intramedullary canal of long bones (Boldrey, 1943; Goldstein and Sturim, 1985; Israel et al., 2018).

Based on this clinical operation, Dingle et al. (2020a) have developed an osseointegrated neural interface (ONI) in a rabbit model that consists of an intramedullary array capable of both efferent and afferent signal transmission and demonstrates physiological and histological signs of regeneration over 3 months. Cuff electrodes are renowned as the least invasive, and least selective interfaces on the PNI spectrum, with sieve electrodes at the opposing end of the spectrum, being the most invasive and offering the most selectivity (Navarro et al., 2005). A confounding factor in this justification is that sieve electrodes are typically tested in neurorrhaphy models in rodents, a model that is well known for its robust neural regeneration and return of function of intact distal target organs (Vela et al., 2020), but is not reflective of the amputation paradigm, in which regeneration is typically haphazard in the absence of a distal target, leading to neuroma formation. Dingle et al. (2020a) demonstrated that the robust three-dimensional axonal sprouting associated with neuroma formation can be passively redirected through transit zones of a dummy sieve electrode housed within the medullary canal. Given this evidence, we believe that the invasive procedure of transecting a nerve in order to insert a sieve electrode is a moot point in the amputation setting, as neurotmesis is unavoidable and the true distal targets are lost. Alternatively, we propose that the neural regeneration giving rise to problematic neuromas can be utilized to innervate sieve electrodes and ultimately restore function though a neuroprosthetic.

In this research report we explore the idea and concept of housing a sieve interface within the medullary canal of long bones as part of an ONI as a method for improving selectivity within this model. This sieve is designed to abut the terminal end of residual nerve and capitalize on the axonal regeneration that would otherwise lead to neuroma formation. *The working hypotheses* are that (A) the addition of a sieve interface to a cuff electrode housed within the medullary canal of the femur as part of an ONI would be capable of measuring bi-directional (efferent and afferent) compound nerve action potentials (CNAPs) through a greater number of channels; (B) that signaling improves over time; and C) that stimulation at this interface generates measurable cortical somatosensory evoked potentials (SSEPs) through a greater number of channels. The cuff electrode allows for comparison to historic data utilizing our amputation model and cuff based ONI (Dingle et al., 2020a). The mechanical stability of the bone may help mitigate damage to the interface resulting from micro-motion while simultaneously providing an electrically insulated environment less prone to myoelectric noise. In addition to the favorable neural interface environment, the intramedullary canal enables the osseointegrated percutaneous integration of a prosthetic, which can simplify the percutaneous routing of leads (Ortiz-Catalan et al., 2014).

## Methods

### Experimental Design

This experiment was designed to examine the concept that the medullary canal represents an ideal environment for the stable housing and engagement of a sieve electrode, as part of an ONI. In order to achieve proof of concept we performed a short-term experiment in a single animal wherein modifications were made to the intramedullary array of our previously published ONI (Dingle et al., 2020a) to include radial electrodes with substantial transit zones for the nerve regeneration (MacEwan et al., 2016). The modifications made to the array served as a less-expensive and more rapidly produced model to measure osseointegrated neural function for confirming proof of concept. Successful proof of concept was predetermined as the ability to record efferent and stimulate afferent signals through the sieve interface comparable to those generated through the original cuff component. This modified ONI was implanted in one New Zealand White rabbit (*n* = 1, Female, 2.7 kg), monitored with repeated electrophysiology measurements (18–34 randomized trials per stimulation) at four time points over a 12-week period to determine nerve engagement with the sieve electrodes and confirmed by complementary engagement of the cuff electrode. Engagement of the cuff was qualitatively compared to historic data (Dingle et al., 2020a). Histological assessment of regeneration was attempted, however, tissue was substantially damaged during retrieval from within the bone. This study was not intended to demonstrate the preferential function of any specific sieve interface design; rather, the intention was to demonstrate proof of concept for stably housing a sieve interface within the medullary canal in an amputation model, supporting rigorous studies of more advanced electrodes toward clinical applicability. All animal procedures were approved by the University of Wisconsin Institutional Animal Care and Use Committee (IACUC, #V005256), the United States Army Medical Research and Material Command (USAMRMC, #DARPA-8728), and the Animal Care and Use Review Office (ACURO, #DARPA-8728).

### Device Fabrication

#### ONI Device

**FIGURE 1:**
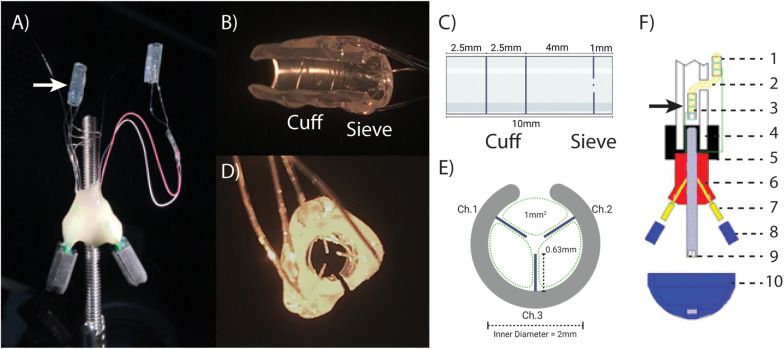
The osseointegrated neural interface (ONI): **(A)** Photograph of the implanted ONI, with a modified intramedullary array (white arrow), containing an additional sieve interface. **(B,C)** A photograph and schematic, (respectively), of the modified intramedullary array, showing the placement of cuff and sieve electrodes. **(D,E)** A photograph and schematic, (respectively), of the sieve electrodes highlighting transit zones (green dotted lines, 1 mm^2^) around the electrodes and channel designation, representative of the implanted electrode. The cut along the silicon tubing visible in **(B,D,E)** is to enable implantation around the nerve. **(F)** A schematic diagram of an implanted ONI highlighting the placement of the modified intramedullary array (black arrow) as well as the proximal array (1), nerve entering bone via the corticotomy (2), intramedullary array consisting of a cuff and sieve (3), bone (4), bone cement (5), epoxy resin (6), PCB (7), PCB cap (8), intramedullary screw (9), cap to protect PCBs (10). Green lines indicate Pt/Ir wire used to create electrode contacts (1 and 2), and connect to independent PCBs (7).

The ONI device (Figure 1) consists of two arrays constructed from silicon cuff tubes with embedded electrodes, each connected to a printed circuit board (PCB, Tucker-Davis Technologies (TDT), Florida, United States) secured to an osseointegrated, percutaneous screw (M4 × 50 mm cup point grub screw, ACCUGROUP, Huddersfield, United Kingdom) (Dingle et al., 2020a). In addition to basic cuff electrodes, three platinum iridium wires (Pt/Ir) with 1mm of insulation removed were placed through the distal end of the intramedullary cuff extending radially into the lumen to create a sieve electrode interface, with three 1 mm^2^ transit zones for sub-chronic electrophysiological interrogation of axonal engagement (Figures 1B–E). The sieve electrodes were based on previously published designs to maximize transit zones (MacEwan et al., 2016), and only added to the distal intramedullary array that interfaces with the terminal end of the amputated nerve within the bone (Figure 1F). The proximal array, connected via insulated Pt/Ir wire, is interfaced outside of the bone and required to perform stimulation and recording of CNAPs under anesthesia. Arrays consisted of silicone tubing (A-M Systems silicone tubing, 1.98 mm inner diameter, 0.6 mm wall thickness) and Pt/Ir wires (A-M Systems 125 μm Pt/Ir PFA coated wire). For the proximal array, tubing was 7.5 mm in length, electrode wires placed at 2 and 4.75 mm apart from the center. For the intramedullary array, tubing was 10 mm in length, cuff electrodes placed at 2.5 and 5 mm with sieve electrodes placed 1mm from the distal end (Figures 1B,C). All electrode wires were secured with quick setting silicone (Kwik-Sil, World Precision Instruments, Sarasota, Florida, United States). The proximal array was connected to the PCB via 7cm of Pt/Ir wire, while the intramedullary array was connected to a separate PCB via 4cm of Pt/Ir wire. Both PCBs and Pt/IR wires were affixed to the percutaneous screw with UV curable dental acrylic (Flow-It Accelerated Light Cure, PENTORN, Orange, CA, United States).

#### Micro-Electrocorticography Array

A custom 16-channel Micro-electrocorticography array (μECoG) array was fabricated using a previously described photolithography lift-off process (Thongpang et al., 2011). The array measured 4 mm × 4 mm with 16 circular platinum electrodes in a 4 × 4 grid spaced 500 μm apart on parylene. Each electrode measured 200 μm in diameter.

### Surgical Procedures

#### Surgical Creation of the ONI

The surgical creation of an ONI consists of a transfemoral amputation and subsequent implantation of the ONI device in New Zealand White rabbits as previously described (Dingle et al., 2020a). Prior to surgery animals were given one dose of Combi-Pen 48 antibiotic (500,000 IU/kg SQ. Bimdeda, Oakbrook Terrace, IL, United States). Rabbits were induced with ketamine (10–15 mg/kg IM), midazolam (0.5–2.0 mg/kg IM), and inhaled isoflurane (0–5% in 100% oxygen). After general anesthesia was reached the anesthetic plane was maintained through isoflurane (0–5% inhaled with O_2_) Vitals including O_2_ and CO_2_ saturation, temperature, blood pressure, and heart rate were monitored through the duration of surgery (Cardel Veterinary Monitor 9,500 HD, Midmark, Dayton, OH, United States). A corticotomy is drilled 2 cm above the midpoint of the femur before a mid-femoral amputation is performed. The transected sciatic nerve was threaded through the corticotomy and inserted into the intramedullary array so that the terminal nerve end abuts the sieve (Figure 1D). The intramedullary array is secured to the nerve with an epineurial stitch and both are pressed back into the medullary canal, followed by the intramedullary screw and secured with veterinary bone cement (BioMedtrix, Boonton, New Jersey, United States). The proximal cuff was then wrapped around sciatic nerve outside the bone, roughly 3 cm proximal to the intramedullary array, secured with an epineurial stitch and the amputation site closed.

#### Craniotomy

At the experimental endpoint of 12 weeks, a craniotomy was performed under anesthesia over the somatosensory cortex contralateral to the amputation to place a custom μECoG array to record SSEPs in an acute procedure. The craniotomy was initiated near Bregma, creating a window 5 mm by 8 mm, measured by the coronal and sagittal suture, respectively. After the craniotomy and electrode placement were completed the animal was taken off of general anesthesia. Subsequent continuous IV infusion of dexmedetomidine (0.06–0.12 mg/kg/hr) and ketamine (15–25 mg/kg/hr) enabled cortical recordings.

### Electrophysiology Procedures

#### Compound Nerve Action Potentials

In this experiment, we aim to address the working hypotheses that (A) the addition of a sieve interface to a cuff electrode housed within the medullary canal of the femur as part of an ONI would be capable of measuring bi-directional (efferent and afferent) CNAPs through a greater number of channels, and (B) that signaling improves over time. Electrophysiology was performed at weeks 3, 5, 8, and 12 post-implantation under general anesthesia. Printed circuit boards (PCBs) percutaneously connected to implanted electrodes were connected to a TDT system via a zero insertion force (ZIF) clip headstage. Prior to each recording session impedance of each electrode was measured at 1,120 Hz using the PZ5 Neurodigitizer (Tucker Davis Technologies). Evoked potentials were recorded using a TDT PZ5 Neurodigitizer with a sampling rate of 25 kHz. CNAPs were generated through monophasic constant current pulses with a phase duration of 30 μs across a range of amplitudes (100 μV–8 mA), this range of amplitudes was used to find the stimulation threshold and confirm a graded response. The resulting CNAPs were low pass filtered (4th order Butterworth corner frequency = 5 kHz) and averaged to identify peak response from each of the stimulation-evoked CNAPs (*n* = 18–35 randomized trials per stimulation amplitude) and averaged across trials. Peak amplitude measurements were taken at 3 and 5 weeks and compared at 5 mA herein. Direction of stimulation was controlled through stimulation of the proximal or intramedullary array. Stimulation via the proximal array and recorded through the intramedullary array prompted efferent CNAPS. Conversely, afferent CNAPs were prompted through stimulation of intramedullary array and measured by the proximal array. Stimulations and recordings with the intramedullary array were performed sequentially though the cuff and each of the three sieve electrodes.

#### Somatosensory Evoked Potentials

In this experiment, we aim to address the working hypothesis (C) that stimulation at this interface generates measurable cortical SSEPs through a greater number of channels. The capacity to elicit somatosensory potentials from a sieve interface via an ONI was tested in an acute experiment at 12 weeks post-surgery. Stimulation was delivered to the terminal end of the nerve via the intramedullary array, stimulating through the cuff and each of the sieve electrodes separately. Responses evoked by stimulation were recorded via μECoG placed over somatosensory cortex contralateral to the amputation. Charge balanced single biphasic pulses (amplitude 100 μA–8 mA, phase 30 μs) were applied to confirm graded responses using an A-M Systems Isolated Pulse Stimulator Model 2100, controlled by a TDT Rz2 BioAmp Processor which was connected to a PCB on the μECoG via ZIF clip. Cortical responses evoked by stimulation from 30 randomized trials (*n* = 30) were amplified (×2), bandpass filtered (corner frequencies: 2.2 Hz–2.7 kHz) and digitized at 6 kHz using a PZ5 Neurodigitizer (TDT) (Dingle et al., 2020a).

### Statistics

A total of seven data points were extracted around the time of peak response from each CNAP post filtering and averaged across time and trials in order to quantify the neural response. A two-way ANOVA with Tukey-Kramer *post hoc* test was performed on CNAP peak to peak response as evoked by stimulation amplitude for each of the recording electrodes (cuff, sieve electrode 1–3). All electrophysiological data analysis was performed in Matlab (Mathworks).

## Results

This work demonstrates proof of concept that a sieve interface can be housed and function within the medullary canal as part of an ONI. The previously published cuff based ONI (Dingle et al., 2020a) was successfully modified to contain an additional sieve interface (Figure 1). This modification consisted of three radial electrodes to form a sieve electrode based on previously published designs to combine both sieve and cuff interfaces (Kim et al., 2020) and maximize transit zones (MacEwan et al., 2016). The modifications did not impact surgical implantation and the animal recovered well and remained unremarkable throughout the 12-week experimental period.

### Electrophysiology

#### Compound Nerve Action Potentials

**FIGURE 2:**
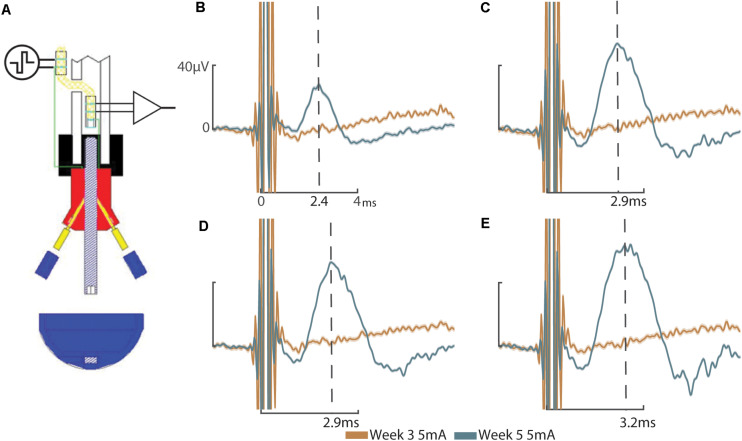
Compound nerve action potentials: **(A)** Schematic representation of efferent stimulation through the ONI demonstrating application of electrical stimuli to the nerve via the proximal cuff and measured evoked potentials using the intramedullary cuff. Efferent CNAPs evoked from stimulation of the proximal cuff where recorded through the cuff and each of the sieve electrodes. **(B)** CNAPs recorded through the cuff at week 3 (peak amplitude = 0.82 ± 2.5 μV) and week 5 (peak amplitude = 27.72 ± 0.88 μV). **(C)** CNAPs recorded through Sieve Ch. 1 at 3 weeks (peak amplitude = 0.78 ± 3.61 μV) and 5 weeks (peak amplitude = 53.78 ± 0.64 μV. **(D)** CNAPs recorded through Sieve Ch. 2 at 3 weeks (peak amplitude = 2.91 ± 1.04 μV) and 5 weeks (peak amplitude = 52.60 ± 0.59 μV). **(E)** CNAPs recorded through Sieve Ch. 3 at 3 weeks (peak amplitude = 3.20 ± 1.12 μV) and 5 weeks (62.40 ± 1.30 μV). Peak amplitude at 3 weeks was determined manually, based on the timing of peak amplitude at 5 weeks, indicated by the dotted lines with times annotated on the *x*-axis **(B–E)**. Stimulation at 5 mA shown. Uniform scale bars **(B–E)** depict 0–40 microvolts (μV, *y*-axis) and 0–4 ms (ms, *x*-axis) as annotated in **(B)**. Standard deviation is shown underlying they average response **(B–E)**.

**FIGURE 3:**
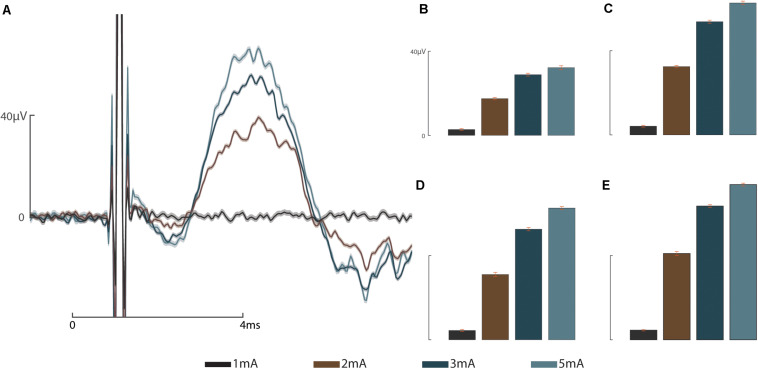
Graded response to stimulus: **(A)** Representative graded response to stimulation at 1, 2, 3, and 5 mA from sieve Ch. 3. **(B)** Graded response to stimulation recorded by the intramedullary cuff electrode. **(C)** Graded response to stimulation recorded through sieve Ch. 1. **(D)** Graded response to stimulation recorded through sieve Ch. 2. **(E)** Graded response to stimulation recorded through sieve Ch. 3. Scale bars depict 0–40 microvolts (μV, *y*-axis) as annotated in **(A,B)**, and 0–4 ms (ms, *x*-axis, **A**).

These results address the working hypotheses that (A) the addition of a sieve interface to a cuff electrode housed within the medullary canal of the femur as part of an ONI would be capable of measuring bi-directional (efferent and afferent) CNAPs through a greater number of channels, and (B) that signaling improves over time. At week three neither the cuff (impedance = 3.8 kΩ, peak amplitude = 0.82 ± 2.5 μV) nor the sieve electrodes (impedances = 13.6, 13.9, and 9.5 kΩ, peak amplitudes = 0.78 ± 3.61 μV, 2.91 ± 1.04 μV, and 3.20 ± 1.12 μV for channels 1–3, respectively) were able to successfully engage the nerve. Given another 2 weeks (week 5) engagement was achieved across all channels demonstrating improved electrophysiological function of the nerve in bone. At week 5, efferent responses are not equal across all channels, demonstrating a two-fold increase in peak amplitude response from the sieve electrodes (impedances = 13.0, 15.1, and 9.2 kΩ, peak amplitudes = 53.78 ± 0.64 μV, 52.60 ± 0.59 μV, and 62.40 ± 1.30 μV for channels 1–3, respectively, average peak amplitude = 56.26 μV) compared to the cuff (impedance = 2.4 kΩ, peak amplitude = 27.72 ± 0.88 μV) (Figure 2). Statistical analysis revealed a significant main effect of stimulation amplitude (*F*_3_,_245_ = 902.51, *P* = 4.49e^–132^) and electrode (*F*_3_,_245_ = 178.98, *P* = 1.88e^–61^) on the magnitude of evoked CNAPs, demonstrating consistently higher amplitudes through all three sieve electrodes compared to the cuff, and one sieve electrode (Ch. 3) above all others (Figure 3). A confounding factor in this experiment was that the proximal array was lost due to visibly broken wires found at the percutaneous site at the week 8 recording session. The broken wires were confirmed as an open circuit with impedance testing (≥536 kΩ) and the irreparable nature was confirmed via x-ray. While this array is not considered an essential component of a true ONI, it is required for conducting stimulation and recording under anesthesia. Afferent CNAPs were not successfully recorded.

#### Somatosensory Evoked Potentials

These results address the working hypothesis (C), that stimulation at this interface generates measurable cortical SSEPs through a greater number of channels. The intramedullary array remained functional throughout the sub-chronic 12-week period, as demonstrated by the ability to elicit SSEPs through the cuff electrode and each of the sieve electrodes (Figure 3). Nerve engagement through the three sieve electrodes varies from channel to channel, though statistically relevant spatiality between electrodes cannot be concluded. The strongest responses were recorded by μECoG electrodes 4, 7, and 8 (Figure 3). The three sieve electrodes demonstrate similar waveforms, all of which are greater than responses achieved through the cuff. Spatiality can be determined in the sense that stimulation elicits a response in some cortical regions (e.g., Ch. 4) and not others (e.g., Ch. 16).

## Discussion

Out of the myriad of potential peripheral nerve interfaces (PNIs), those with greater selectivity are considered to be the most invasive, which results in the shortest longevity and stability (Navarro et al., 2005; Ghafoor et al., 2017). Sieve electrodes enable close contact with individual nerve bundles or even individual fibers within the perineurium, resulting in greater selectivity than can be achieved with extraneural PNIs (MacEwan et al., 2016; Urbanchek et al., 2016). This increased selectively can ultimately translate to improved prosthetic control (Tyler and Durand, 2002; Ghafoor et al., 2017; Straka et al., 2018). The downside of such intimate contact is the potential for damage resulting from micro-motion (Branner et al., 2004; Desai et al., 2017; Wurth et al., 2017). Additionally, the classification of sieve electrodes as most invasive is based on the neurorrhaphy model in which they are tested (Navarro et al., 2005) and are not necessarily reflective of the amputation setting. We have previously developed a trans-femoral amputation model in rabbits based on the clinical transposition of nerve into bone to treat symptomatic neuroma that serves as the basis for creating an ONI for prosthetic control (Israel et al., 2018; Dingle et al., 2020b).

In this proof of concept study, we explore the concept of securely housing sieve electrodes in the medullary canal as part of an ONI as a potential method for increasing selectivity. By abutting sieve electrodes to the terminal end of the amputated nerve we demonstrate neural engagement improves over time comparable to a cuff electrode in the same animal (Figure 2) and historic data (Dingle et al., 2020a). Sieve electrodes demonstrated greater neural engagement (two-fold) than cuff electrodes (Figure 2). Furthermore, statistical analysis reveals that each of the sieve electrodes records a significantly higher magnitude than cuff electrodes (Figure 3), demonstrating resistance of the epineurium (Yoshida et al., 2000; Branner et al., 2004; Ghafoor et al., 2017). Histological examination of regeneration through transit zones was attempted, but failed due to tissue damage when retrieving the interface from the osseointegrated hardware within the bone. Neural regeneration through transit zones has been demonstrated previously in this model in the absence of osseointegration and associated electrode hardware. The loss of the proximal array after the fifth week precluded electrophysiological time points matching our previous studies, including the recording of afferent CNAPS from 8 weeks post-operatively (Dingle et al., 2020a).

**FIGURE 4:**
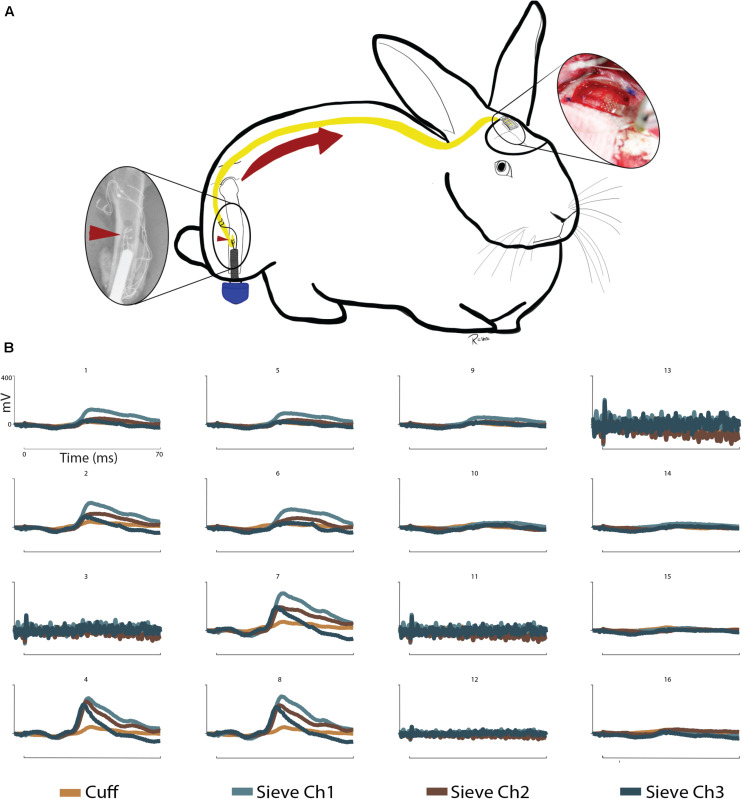
Somatosensory evoked potentials: **(A)** Schematic representation of sensory information written to the CNS via a sieve interface as part of an ONI. Left to right: The intramedullary array (red arrowheads) stimulates the sciatic nerve within the medullary canal of the femur. Afferent action potentials travel from the PNS to the CNS (red arrow) and are recorded over the contralateral somatosensory cortex via μECoG. **(B)** SSEPs recorded via 16 channel micro-electrocorticography in a terminal operation at week 12. Stimulation was provided through the cuff and sieve electrodes. Stimulation results in cortical spatiality as demonstrated by clear signals in channels 4, 7, and 8 from the sieve electrodes and weaker responses from the cuff. Reduced cortical responses can be seen in channels 1, 2, 5, 6, and 9 and no clear signals in channels 10, 14–16. Channels 3, 11, 12, and 13 of the μECoG were not functional at the time of recording. Stimulation at 5 mA shown. Uniform scale bars (channels 1–16) depict 0–400 mV (mV, *y*-axis) and 0–70 ms (ms, *x*-axis) as annotated in B1.

Despite the complication of losing the proximal array after 5 weeks, the intramedullary array remained operational and viability was confirmed by the recording of afferent cortical responses to stimulation 12-weeks post implantation (Figure 4). Cuff and sieve electrodes demonstrated nerve engagement with signals propagating to cortical brain regions. The strongest responses were recorded by μECoG electrodes 4, 7, and 8, (Figure 4). Sieve Ch. 3 had the strongest efferent CNAPs in week 5 (Figures 2, 3) but the weakest afferent SSEPs through to the cortical regions in week 12 (Figure 4). In cortical regions of stimulation, the similarity in the waveforms indicates sieve electrodes are activating similar cortical regions signifying selectivity between these electrodes is poor. This is expected given the rudimentary construction of the array. Spatiality of the cortical response can be observed in the sense that responses to cuff and sieve electrodes differ enough to indicate each electrode simulates different areas of cortex with substantial overlap (Figure 4). As with CNAPs, SSEPs evoked through stimulation of the sieve demonstrated greater peak amplitudes than those achieved with the cuff, supporting the requirement for lower stimulation thresholds with more intimate neural contact (Tyler and Durand, 2002; Ghafoor et al., 2017).

This study was *not* intended to demonstrate the preferential function of any specific sieve interface; rather, the intention was to demonstrate proof of concept for stably housing a sieve interface within the medullary canal and engaging with transposed nerves in an amputation model based on a clinical procedure for treating neuroma (Israel et al., 2018). The ONI model for peripheral nerve interfacing and prosthetic attachment is amenable to the application of more sophisticated sieve electrodes capable of greater selectivity for improved prosthetic control. Our simplified design demonstrates the capacity for bi-directional (efferent CNAPs and afferent SSEPs) stimulation and recording aided by percutaneous connectivity of an osseointegrated prosthetic abutment. Our results support the ability for osseointegrated prosthetics to handle percutaneous neural interface connections, confirming the stability of routing wires (Ortiz-Catalan et al., 2014).

## Conclusion

This proof of concept experiment successfully demonstrates the working hypotheses that (A) sieve interface can be housed within the medullary canal of long bones in conjunction with a cuff electrode as part of an ONI capable of recording efferent CNAPs from a greater number of channels, (B) that the signal improves over time; and (C) that afferent stimulation through the sieve can generate SSEPs, demonstrating the ability to elicit SSEPs through a greater number of channels, contributing to the literature concerning osseointegrated neural interfacing. The ONI allows the application of a sieve electrode on the terminal end of amputated nerves, secured within the bone with direct connectivity afforded by percutaneous osseointegration. This proof of concept supports future work for the application of more sophisticated sieve electrodes within the medullary canal as part of an ONI for improved prosthetic control.

## Data Availability Statement

The raw data supporting the conclusions of this article will be made available by the authors, without undue reservation.

## Ethics Statement

All animal procedures were approved by the University of Wisconsin Institutional Animal Care and Use Committee (IACUC, #V005256), the United States Army Medical Research and Material Command (USAMRMC, #DARPA-8728), and the Animal Care and Use Review Office (ACURO, #DARPA-8728).

## Author Contributions

SP, AD, JW, AS, and MM: conceptualization. AD, JPN, JN, and AM: data curation. AD, JPN, JN, AS, and AM: data analysis. SP and JW: funding acquisition. AD, JPN, JN, BN, YL, AM, and RM: investigation. SP, YL, BN, AD, JN, JPN, AS, JW, MM, RM, and AM: methodology. AD and BN: project administration. SP, JW, MM, and BN: resources. AS, JPN, and JN: software. SP, JW, MM, BN, and AD: supervision. AD and AS: validation. AD, JPN, JN, and AS: visualization. AD and AM: writing – original draft preparation. AD, JPN, AM, and RM: writing – figures. AM, AD, JPN, JN, WZ, YL, RM, BN, MM, AS, JW, and SP: writing – review and editing. All authors contributed to the article and approved the submitted version.

## Conflict of Interest

JW has ownership interests in NeuroOne Medical Inc., and Neuronexus Technologies, companies which produce and market micro-electrode technologies, including peripheral neural interfaces. JW and SP own intellectual property related to the material discussed in this manuscript which has been assigned to theWisconsin Alumni Research Foundation. The remaining authors declare that the research was conducted in the absence of any commercial or financial relationships that could be construed as a potential conflict of interest.
